# Dental Army Service in the Philippines

**Published:** 1907-06-15

**Authors:** S. D. Boak

**Affiliations:** U.S.A. Dental Surgeon


### Dental Army Service in the Philippines.

BY S. D. BOAK, U. S. A. DENTAL SURGEON.

At the time I first went to Manila, there were in that city sixteen Filipino dentists, five Spaniards, one Englishman, five American and two Chinese. One of the Filipinos was a woman, and the Englishman was an advertiser. The American offices were well equipped with up-to-date furnishings and appliances, but the native offices were meagerly equipped. There is no dental depot in Manila, but an accommodating English drug store supplies gold plate and solder at from $2.50 to $3.50 per pennyweight, and gold cylinders at $8.00 for an eighth of an ounce. Other things at proportionate rates. The native Filipino is a great coward when it comes to having his teeth filled; he prefers to let them decay and lose them.

My first assignment for duty was at San Fernando, about 40 miles from Manila. My first experience after arriving there was to get settled in a two-room shack, which was destroyed by a terrific rain storm and typhoon just as I had gotten my instruments and appliance well settled. I found in the town a native dentist who spent most of his time making ornaments for the natives out of silver.

That caries progresses more rapidly in tropical climates is an assured fact, especially among the Anglo-Saxons, and from observations it may be attributed to the following causes, all things being equal.

1st. Lowering of the vitality, and a lessening of the resisting powers of the body.

2nd. Acidity of the oral secretions. I tested 1,500 men's saliva, not only as a combination of the secretions of the parotid, submaxillary, sublingual and racemose glands, but tested them separately as they issued from their ducts. Result of the 1,500 cases: Fifty per cent. acid reaction; 30 per cent. slightly acid or neutral; 10 per cent. neutral; 10 per cent. alkaline.

3rd. Active campaigning, scant water supply, entire time filled with martial duties, constant alertness for the enemy, and in self-preservation, with loss to appear anything but the real fighting machine, this being especially true of the troops stationed in Mindanao and the Sulu archipeligo, amongst the Moros, which rendered it almost impossible for a soldier to give even the ordinary care to his person, much less hygienic attention to his mouth. To these conditions add the trouble to secure a soldier after beginning treatment; when sent for he is either out on the firing line, doing guard or old guard fatigue duty, on a scouting expedition, or temporarily attached to some other outfit; or about the time you were in the midst of a month's visit to some command, be ordered away, or the command get field orders; supplies fail to reach you, due to lack of transportation by water or land, leaving you to complete a two months' detail withont amalgam, cement, nerve broaches, or other necessary supplies, necessitating the use of mummifying paste, sealed in with cotton and sandarac varnish or gutta percha, where pulps have been extracted. It was often a serious question when out of supplies just what to do to secure permanent relief from pain and suffering, the dental surgeon being thrown on his own resources and ingenuity to accomplish it. A conscientious professional man will not extract except as a last resort, especially if he sees a chance of saving teeth that would otherwise be sacrificed.

Let me put before you a typical case. I was ordered to go through a battalion of infantry, 450 men in the battalion, in one month, men being brought to the office in squads of twenty under a non-commissioned officer. Upon examining a soldier, find on left side one or two of the superior or inferior molars or bicuspids missing. Right side a molar or a bicuspid, or both, broken down and necrosed, this side bearing the whole stress of mastication. The question is, shall we extract them, leave them in that condition, or begin treatment, knowing the difficulties that will handicap us in the conservative treatment of such cases. If we extract we save our future reputation and take no chances, for if the soldier has trouble, not understanding the conditions or disadvantages we labor under, and has to suffer with no hopes for relief in sight, we get damned for it; for over there it is not a case of getting on a train or boat and in an hour or so get relief, but in most cases it means days. If we make some excuse and put them off, in all probability they will begin to give trouble at a very inopportune time, and a kick is registered by the commanding officer of the company, that a dental surgeon visited that post and left before he finished his work. This being forwarded through military channels, everybody from the C. O. up gets a chance to express their opinion, favorable or otherwise, as the case may be, and in all probability you are called upon to explain why treatment was not furnished in certain cases. If we start in to treat, and when ordered away find root canals are not in condition to fill, we have to place a dressing in the roots, insert cement and trust to nature to do the rest. This also applies to fillings. In the great majority of cases, especially during 1901 and 1902, it was impossible to get a man for a second sitting to polish off work, consequently many of these men came home, and falling into the hands of the civil practitioner, our work was criticised, when they did not know the conditions the dental corps were laboring under. This gives you some idea, from actual experiences, what we have been up against. Many of us also having to operate in tents with poor light. I speak of these conditions and causes to let you know how the army practice differs from the civil, for you must remember in your practice, patients come of their own accord in most instances and help you to succeed in your care of them. In the military practice we examine the men as a body and do the required work whether they wish it or not, and the fact of men being ordered to have dental work done, independent of their own desires in the matter is not calculated to secure their co-operation, as general orders require that any soldier unfit for duty, from a cause that is removable by an operation not endangering life, who refuses to be operated upon, will be tried by court-martial and punished.

During my two months' stay at Tarlac, I noticed a peculiar custom that might interest you. One evening I went to a native dance and noticed a young girl who had what appeared to be a gold crown on her right superior central incisor. I paid little attention to the fact at the time, but the next day passed her on the street and noticed that her sister had a crown on the same tooth, and she had none. About a week later I noticed that the sister was minus her crown and that the mother was sporting one. I watched this seeming interchanging for over a month, until one day a favorable opportunity presented itself, and I asked the original owner what kind ot a crown that was whcih she wore the night ot the dance. She replied, "bueno custombre" meaning very good custom, and told me that she would show it to me if I would come to the house. That night after supper I was on deck bright and early, and tound the mother still the proud possessor of the golden headlight; it was simply a facing of gold to fit the front of the tooth, with two wings attached that were passed through the interdental spaces on either side of the central and bent on the lingual aspect of the central to secure it in position. This custom I found quite prevalent in other provinces, especially of southern Luzon.

All the Moros chew betel nut, mixed with a paste composed of iron filings and cocoanut milk. The betel nut (Bonga) is the seed of the betel palm, a slice of which is wrapped in the leaf of the Buyo plant. On this leaf, before wrapping it around the betel nut, is smeared a paste of lime and water; this is the Pilipino's substitute for chewing tobacco; however, the Filipino does not add the iron filings and cocoa milk as the Moros do; this latter mixture causes an oxide of iron to be deposited on the teeth, which in time encrusts them, making the teeth black. When the children reach the age of puberty, the Moros file their teeth with a rat-tail file convexly, which gives them a scooped-out appearance; this filing, of course, goes into the dentine, and one would suppose would be very painful, but I have seen them doing it in all stages, and they do not seem to suffer the slightest discomfort from the operation. After the teeth take on the black appearance the children are married according to the wishes of their parents; this applies to both sexes.

Across the Basilan Straits from Zamboanga, and in plain sight, is the island of Basilan, which is inhabited in its interior by the Yakans, a very primitive race of Moros, who wear only the breech-clout and let their hair grow long. Exceedingly fierce both in looks and disposition, these Moros file their teeth to a point; the teeth in the front of the mouth having an appearance of a saw blade. They are head-hunters, as are also many of the other tribes inhabiting the interior of Mindanao, and they endeavor to make their features as disgusting and frightful as possible for the purpose of designating their ideas, and for scaring and confusing their foes when they meet them in combat; and you certainly see some frightful-looking mouths.

As to dentistry among them, they have none other than the filing of their teeth, but the Moros that live in the coast towns and have been brought into contact with other races have a crude system of it. They fill teeth by hammering and wedging small pieces of brass wire, on the same principle as we work non-cohesive gold; also cut out pieces of ebony as near the size of the cavity as they can, and pound it in, making not even a pretense of cleaning out the decayed part. I also have in my possession two pieces of their bridge-work; the teeth were cut off and the pin used to hold the piece in place was wrapped with a piece of thread and pushed into the root eanal, where the moisture caused it to swell and hold it in place. If they have an exposed pulp or any other trouble, they patiently stand it until nature adjusts the matter. The Lake Lanao Moros also have a custom of taking the teeth of their enemies slain in battle, and either setting the crown in a ring with the roots standing up, or splitting the tooth in half and using part of it for a setting; this can better be illustrated by the one I will pass around for your inspection.

### CHINESE PROFESSIONAL MEN.

The consulting room of the Chinese practitioner of dentistry is a booth or tent at a street corner or in a court of a temple; you know it by the long strings of extracted teeth that hang up. On one string I noticed two pieces of jaw bone that were not very re-assuring.

The medical man has dried snakes and roots, curious herbs and out-of-the-common dried fruits. Their walls are stuck over with plasters that have done their duty and been returned by grateful patients in testimony of their efficiency. Outside of these tents or shops is always a crowd, hoping to see an operation, and no doubt these idlers help patients to bear without a moan, and often with sweet smiles, the burning, nipping, punching, puncturing and other tortures, which, with simple faith, they pay to have inflicted upon them. In China the distinction between a physician and a surgeon, I am told, is drawn by calling the former an outside doctor and the latter an inside one. An example to illustrate: I was told by a Canton Chinaman that a relative, in fighting pirates on the West river, had an arrow shot through his arm. An outer doctor was called, who cut off the two ends of the arrow and put a plaster over the wound. "But," the patient said, "the rest of the arrow is still there; "that," answered the medico, "is not my business; you must go to an inner doctor if you want it removed." Confucious said, "It is the duty of every man to return his body to the earth as whole as when it came from his mother's womb." In deference to this dictum, the Chinese medical men do not dissect dead subjects, and are, in consequence, ignorant of the location of the largest viscera.

The genital organs of a cat are given to promote virility; for diseases of children centipedes are prepared. Toads' eyebrows are used to provoke sneezing, and thus clear the head. For sick stomach, earth worms rolled in honey are swallowed alive. Other medicines, infusions from bones of tigers for strength, cockroach tea, fungus grown upon coffins, shavings of rhinoceros' horn. A missionary told me he was called to see a man suffering from fits; found him on his back on the ground, smelling white mice in a cage, with a dead fowl strapped to his chest and a bundle of dried grass strapped to the soles of his feet. While at Shanghai, I joined a crowd in a temple court and saw a man acapunctured for a swollen leg. About a dozen needles, like sewing-machine ones, only longer, were driven into the limb; on the top of each needle was fastened oiled tow or some other flammable material and lighted; when the needles became redhot the flesh began to sizzle as though a beefsteak were being cooked. Superior to pain, the patient chatted and laughed with the crowd. In Canton I saw a Chinese dentist extract a bicuspid tooth for his patient with the thumb and forefinger of his right hand, whilst an admiring crowd looked. This dentist wore around his neck a ghastly string of teeth as testimonials of his skill; he also had some crude forceps, which looked more like blacksmith's pinchers, they being used when his dirty fingers were of no avail. The Chinese believe that when they get the toothache, a small worm in the tooth is the cause of it; this idea is kept alive by the Chinese dentists, who palm a small maggot, which they keep in a box to show the patient after extraction, for which they charge extra for securing.

Since the American occupation of the Philippines, a dental law was passed by the Commission. The law requires the appointing of three reputable practitioners of dental surgery, who shall be graduates in good standing of legally incorporated dental educational institutions recognized by the National Association of Dental Examiners of the United States of America. The members receive as compensation $2.50 for each candidate examined, the secretary-treasurer receiving $150 per year.

